# Natural Ventilation Reduces Cooking-Related PM_2.5_ Peaks Indoors

**DOI:** 10.1021/acsestair.5c00427

**Published:** 2026-01-29

**Authors:** Yizhou Su, Yuqing Dai, Zongbo Shi, Yirui Jiang, Lingchen Kong, Christian Pfrang

**Affiliations:** † School of Geography, Earth and Environmental Sciences, 1724 University of Birmingham, Edgbaston, Birmingham B15 2TT, U.K.; ‡ Department of Computer Science and Technology, University of Cambridge, Cambridgeshire CB3 0FD, U.K.; § Department of Architecture, University of Cambridge, Cambridge CB2 1PX, U.K.; ∥ Department of Meteorology, University of Reading, Whiteknights, Earley Gate, Reading RG6 6BB, U.K.

**Keywords:** Indoor air quality, cooking emissions, ventilation, PM_2.5_ exposure

## Abstract

Indoor cooking generates intense, short-duration fine particulate matter (PM_2.5_) peaks with acute health risks. To quantify the efficacy of natural ventilation configurations, we conducted approximately two months of continuous monitoring in a modern UK one-bedroom apartment, comparing three ventilation scenarios during cooking: fully opened (all windows and internal doors open), door-opened only (internal doors open but windows closed), and fully closed (all windows and internal doors closed). Air quality sensors were calibrated against a reference instrument (Fidas 200E) both before and after the field deployment. During the study period, outdoor PM_2.5_ mass concentrations ranged from 0.4 to 31.0 μg m^–3^, averaging 6.3 μg m^–3^. Indoor concentrations were substantially higher than average outdoor levels, with the fully opened scenario yielding the lowest exposure at 14.9 μg m^–3^ in the living room/kitchen and 15.4 μg m^–3^ in the bedroom. Relative to the fully opened scenario, PM_2.5_ concentrations increased by 58.4% (living room/kitchen) and 55.8% (bedroom) under door-opened only conditions, and under fully closed conditions by 28.9% and 27.9%, respectively. These findings demonstrate that simultaneous opening of windows and internal doors during cooking can substantially reduce acute PM_2.5_ exposure, offering a simple, low-energy strategy to mitigate short-term health risks in naturally ventilated apartments.

## Graphical abstract

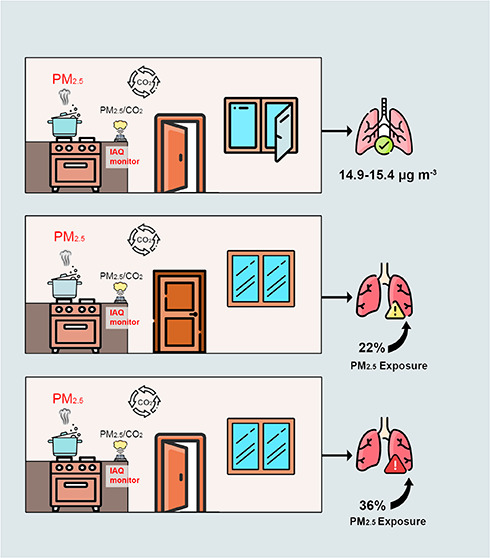

## 1 Introduction

Indoor air pollution is a major contributor to the global burden of disease.^1–4^ According to the World Health Organization’s (WHO) 2024 update, indoor air pollution is associated with approximately 3.2 million premature deaths annually, including over 237,000 among children under five, with fine particulate matter (PM_2.5_) identified as a key pollutant linked to adverse health outcomes.^5^ Short-term elevations in PM_2.5_ concentrations have been linked to increases in emergency respiratory admissions, cardiovascular events, and all-cause mortality; older adults (≥65 years), particular males, are particularly vulnerable.^6–10^ Although much research and many mitigation efforts have focused on biomass combustion in low- and middle-income countries, emerging evidence shows that substantial indoor PM_2.5_ exposures also occur in modern dwellings across high-income settings. Notably, even in homes without indoor combustion (e.g., using electric hobs), cooking can generate high PM_2.5_ concentrations from food and oil aerosols. In homes using gas for cooking, these aerosol emissions are further compounded by combustion byproducts.^11^

In homes without continuous mechanical ventilation systems (e.g., whole-dwelling supply–extract or heat-recovery ventilation), many dwellings across both low- and high-income countries rely on natural ventilation driven by wind and buoyancy, which depends on occupant behaviors such as opening windows and doors. In the UK, natural ventilation remains predominant in many existing residential buildings, often supplemented by intermittent extract fans,^12,13^ but the understanding of indoor pollutant levels in the UK and especially in private homes remains limited, and there is an urgent need for an indoor emissions inventory.^14^ This is largely due to the scarcity of in-home measurements, the heterogeneity of occupant behaviors, and the logistical challenges associated with monitoring private residential environments. Among noncombustion sources in nonsmoking homes, cooking-process emissions are widely recognized as both the most frequent and the most intense contributors to indoor PM_2.5_ in such environments.^15,16^ Field studies have shown that hob frying, grilling, and stir-frying can generate transient PM_2.5_ concentrations up to an order of magnitude higher than simultaneous outdoor levels.^17–19^ These short-term cooking-related episodes have been shown to dominate daily personal exposure profiles in nonsmoking homes,^20,21^ and are implicated in acute respiratory symptoms, particularly among individuals with asthma or chronic obstructive pulmonary disease.^22^ As such, the effectiveness of household ventilation becomes a key determinant of short-term exposure outcomes during cooking events.

Ventilation plays an important role in modulating indoor air quality by removing airborne pollutants and exchanging indoor air with outdoor air, the effectiveness of which depends on ambient outdoor air quality and ventilation conditions.^23–25^ Numerous studies have evaluated mechanical ventilation, including kitchen range hoods and whole-house systems.^19,26–28^ The airflow regimes in mechanically ventilated systems differ fundamentally from those in naturally ventilated dwellings. In natural ventilation, air movement arises from transient buoyancy forces and fluctuating wind pressures that may shift or even reverse direction as outdoor conditions change. Mechanically ventilated systems, by contrast, generate regulated airflow, temperature, velocity, and a stable pressure field. Performance data obtained under such forced-flow conditions therefore cannot be applied to the window- and door-driven scenarios typical of naturally ventilated homes.^29,30^

Some studies have used modeling approaches to simulate combined ventilation scenarios (e.g.,^31^), but empirical data capturing how occupant behaviors influence short-term exposure remain scarce. Experimental studies have also examined cooking-related pollutant emissions under natural ventilation conditions, including the effects of window and door opening (e.g.,^32^). However, such studies are typically conducted in controlled or test-kitchen environments and focus on kitchen-scale conditions, stove location, or thermal comfort, rather than occupant-controlled ventilation behaviors in lived-in dwellings or short-term exposure dynamics across interconnected rooms.

This gap is especially pronounced in modern households with no indoor combustion sources (e.g., using electric hob), where cooking emissions remain prevalent, but ventilation relies entirely on occupant actions. There is still lack of episode-resolved field studies that pair occupant-logged window/door states with cooking-related pollutant metrics (e.g., peak and time-normalized PM_2.5_ concentration); most prior work relies on test kitchens or focuses on mechanical hoods rather than naturally ventilated, occupied dwellings. As a result, the extent to which every day, occupant-controlled natural-ventilation choices during routine cooking reduce (or exacerbate) short-term exposure remains uncertain.

Buildings are responsible for a substantial share of global greenhouse-gas emissions: in 2022 building operations accounted for 30% of global final energy consumption and 26% of energy-related CO_2_ emissions.^33^ The International Energy Agency (IEA) notes that to align with a Net-Zero Emissions target in 2050, energy use in all new buildings and 20% of existing building stock must be zero-carbon-ready by 2030.^33^ Ventilation therefore needs to be considered not only as a health intervention but also as part of a building’s energy balance. Heating, ventilation, and air conditioning (HVAC) systems consume electricity and contribute to indirect emissions, whereas natural ventilation uses wind- and buoyancy-driven flows and can improve thermal comfort and indoor air quality without mechanical energy inputs.^29^ Recent work highlights that optimized natural ventilation can significantly reduce building energy consumption while enhancing occupant satisfaction, indoor air quality, and thermal comfort.^34,35^ Adaptive thermal-comfort approaches, where occupants adjust natural ventilation behaviors and accept wider indoor temperature bands, can further increase the energy-saving potential of natural ventilation. In this context, targeted use of natural ventilation during short-lived cooking episodes can help maintain acceptable indoor air quality and thermal comfort without the energy demands and greenhouse gas emissions associated with mechanical systems, thereby supporting both public health and climate-change mitigation objectives.^34,35^

Motivated by these dual health–climate considerations, we present a field study quantifying how occupant-controlled natural ventilation affects cooking-related PM_2.5_ concentration in a modern UK apartment. We used portable air quality sensors and time-stamped cooking logs to measure concentration under three practical ventilation scenarios. We estimated the magnitude and variability of PM_2.5_ concentration reductions achievable with simple ventilation practices. Our objectives were to (i) compare peak concentration and the rise rate of PM_2.5_ during cooking across the three common ventilation scenarios and (ii) evaluate event-to-event concentration variability time-integrated PM_2.5_ concentration to inform practical guidance for households.

## 2 Materials and Methods

### 2.1 Field Experiment

The field experiment was conducted between 8 July and 28 August 2024 in a modern fifth-floor apartment located near a major traffic corridor in the city center of Birmingham, United Kingdom (Figure S1). The dwelling comprises an open-plan living room/kitchen (R1), a bedroom (R2), a bathroom, and an internal corridor (R3) linking the living room/kitchen to the bedroom (Figure 1). Air exchange between indoors and outdoors occurred solely through single-sided natural ventilation via two top-hung casement windows: W1 in R1 and W2 in R2, while interior airflow was governed by doors D1 (living room/kitchen-corridor) and D2 (bedroom-corridor).

**Figure 1:**
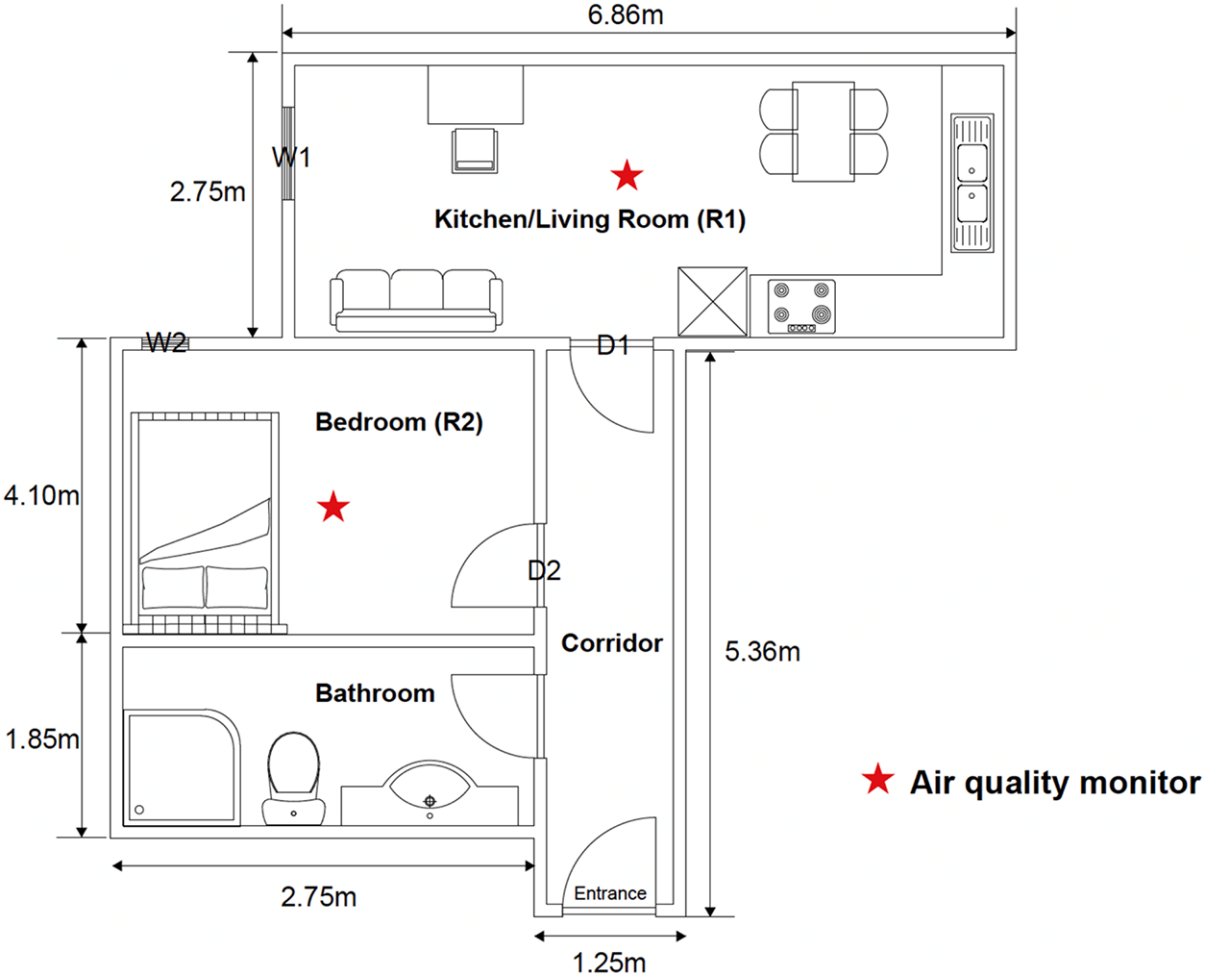
Schematic floor plan of the apartment where the field study was conducted.

The living room/kitchen contains a four-ring induction hob and a recirculating cooker hood with no external exhaust; the hood was not operated during the monitoring period and remained switched off for all cooking events. The bathroom was equipped with a mechanical extract fan, which was also not operated at any point during the campaign.

Outside the defined cooking events, no specific ventilation protocol was imposed. Window and door operation followed normal day-to-day occupant behavior and was not systematically recorded, in order to preserve realistic living conditions and to distinguish this field study from fully controlled experimental simulations. All ventilation scenarios analyzed in this study therefore correspond specifically to the defined cooking periods.

Geometric parameters, including floor area, ceiling height, volume, and effective opening area, are summarized in Table S1. Throughout the campaign, the apartment was continuously occupied by one adult who followed typical daily routines and manually logged the start and end times of each cooking event, all of which took place in the R1 while the occupant remained in R1 throughout each cooking episode. No smoking, candle use, or other combustion-related activities occurred during the monitoring period.

Indoor air quality was recorded continuously with two AirGradient ONE sensors (model I-9PSL; Figure S2), which have also been deployed in large-scale residential indoor air quality monitoring projects, such as the INGENIOUS project in the UK.^36^ The sensors were positioned at breathing height (1.2 m) at the geometric centers of R1 and R2 (see Figure 1), equidistant from ventilation openings (i.e., windows, doors) and the cooking hob. This placement minimized positive bias near the emission source and negative bias near outlets, providing a representative measure of the room-average microenvironment.^37^ Each sensor integrates a SenseAir S8 NDIR sensor for CO_2_ (400–10,000 ppm, ± 40 ppm ± 3% of reading), a Plantower PMS-5003 light-scattering particle sensor for PM_2.5_ (±10 μg m^–3^ below 100 μg m^–3^ or ± 10% above), and a Sensirion SHT-40 for temperature (−40 to +125 °C, ± 0.2 °C) and relative humidity (0–100%, ± 2% RH). All parameters were logged at 5 min intervals.

Continuous indoor air quality monitoring in the living room/kitchen and bedroom was maintained throughout the campaign, while three occupant-realistic natural ventilation scenarios were alternated in a pseudorandom order to minimize time-of-day bias (see Table S2 for exact start–end times and sequence),^38^ specifically:

(1) Fully-Opened (FO): all windows and internal doors open.
(2) Doors-Opened & windows-closed (DO): internal doors (D1, D2) open; windows (W1, W2) closed; and
(3) Fully-Closed (FC): all windows and internal doors closed.

The three ventilation scenarios were selected to isolate two key determinants of indoor pollutant behavior: inter-room connectivity and indoor–outdoor air exchange. The FO scenario represents conditions with both full internal connectivity and active outdoor exchange; the DO scenario isolates internal connectivity in the absence of outdoor exchange; and the FC scenario represents minimal connectivity both internally and with the outdoors. Together, these scenarios bracket common occupant-controlled ventilation states in compact apartments and enable a clear comparison of the relative roles of internal connectivity and outdoor exchange under typical residential conditions.The effective free-opening area, defined as the projected clear area at the fixed opening angle used during testing, is listed in Table S1. The start and end of each cooking episode, along with the ventilation condition in force, were logged in real time and collated in Table S2. Subsequent analysis included only the active cooking period defined by these logs; postcooking decay phases were excluded from exposure calculations to focus on the period of most intense, direct exposure when occupants were actively cooking near the emission source. Cooking tests followed a standardized protocol to ensure consistency across events, comprising typical home dishes including stir-frying, boiling, and steaming of fresh vegetables and lean meat using small amounts of olive oil. The use of heavy salt, oil, or spices was avoided to minimize variability in emission profiles across tests. No heating, cooling, or mechanical exhaust systems were operated during testing, isolating the effects of natural ventilation

Outdoor PM_2.5_ concentrations were obtained from the UK Automatic Urban and Rural Network (UK.-AURN) roadside monitoring station A4540 (UK.-AIR ID: UKA00626; 52.476°N, −1.875°W) on Keeley Street, Birmingham (Figure S1).^39^ The straight-line distance between the studied apartment and the monitoring station is approximately 1.34 km. Hourly data were retrieved from the DEFRA Air Quality Archive and used to characterize the outdoor background during the study period. The A4540 site is classified as a roadside station and is influenced by local traffic emissions, which may lead to higher PM_2.5_ concentrations than urban background locations. However, as the studied apartment is also located adjacent to a major traffic corridor in Birmingham city center, the station was considered representative for characterizing the temporal variability and magnitude of outdoor PM_2.5_ relevant to the study, while recognizing that absolute concentrations may represent an upper-bound estimate of residential outdoor exposure.

### 2.2 Sensor Calibration

Both AirGradient monitors were colocated with a Palas Fidas 200E reference instrument for 14-day periods before and after field deployment to check calibration stability. Calibration comprised simultaneous measurements of PM_2.5_ under three different emission scenarios: clean baseline air, simulated cooking aerosol, and incense smoke, over a range of typical indoor temperatures (T) and relative humidities (RH).^40^ RH and temperature during calibration spanned 48–83% and 5–21 °C, respectively; during the field campaign, they were 38–71% and 21–28 °C. All values were within the manufacturer-stated accuracy of the sensor. A two-segment piecewise multivariate linear regression model, including PM concentration, RH, and T as predictor terms, was subsequently fitted to derive calibration equations;^41^ these equations were used to correct all field data and ensure traceability to the reference method.

For CO_2_ measurements, sensor consistency was evaluated through interdevice colocation, since a reference-grade CO_2_ instrument was not available during testing. The two sensors showed a high degree of agreement, with a near 1:1 relationship (*R* ^2^ = 0.90), indicating good stability and internal consistency (Figure S7). While this approach does not provide an absolute calibration, it allows assessment of sensor stability and agreement. Given that ventilation assessment using CO_2_ focuses on the temporal change in its mixing ratio, such as build-up or decay patterns, rather than on absolute values, this method aligns well with the intended application.^42^ It therefore offers a practical and appropriate alternative in the absence of reference instrumentation.

The detailed calibration procedure, sensor performance evaluation, and quantification of measurement uncertainty are described in the “Sensor Calibration and Performance Evaluation” section of the Supporting Information.

### 2.3 Cooking Exposure Evaluation

To illustrate how ventilation conditions influenced exposure within this case study, we adopted a two-tier metric framework. First, we assessed instantaneous and dynamic exposure using gross peak increment of PM_2.5_ (ΔPM_peak_) and its rise-rate to evaluate pollution build-up. Then, we computed a time-normalized PM_2.5_ dose (*D*_PM,norm_) to further account for differences in cooking duration and better represent exposure intensity per unit time. Meanwhile, observed changes in indoor CO_2_ mixing ratios were used as a real-time proxy for ventilation effectiveness to quantitatively differentiate between the practical impacts of each scenario.

To quantify instantaneous exposure under different ventilation scenarios, we employed a peak concentration increment metric. For each cooking event, the peak increment (ΔPM_peak_, μg m^–3^) was calculated as^19^

1 $$\Delta {PM}_{peak}=\{\begin{array}{ll} C_{peak}-C_{0}, & \mathrm{if strategy}\neq fully‐opened\\ C_{peak}-C_{0}, & if\;strategy=fully‐opened\;and\;C_{peak}\geq {PM}_{out}+\sigma_{noise}\\ 0, & if\;strategy=fully‐opened\;and\;C_{peak}<{PM}_{out}+\sigma_{noise} \end{array}$$

Where *C*_0_ is the baseline indoor PM_2.5_ immediately cooking start time, *C*_peak_ is the maximum indoor concentration during the event, PM_out_ is the concurrent outdoor PM_2.5_ from the Birmingham city-center air quality monitoring station, and σ denotes the sensor noise standard deviation. The sensor noise standard deviation (σ) was estimated as the standard deviation of PM_2.5_ concentrations during stable indoor background periods without identifiable indoor emission sources, representing short-term instrumental variability and background noise. This piecewise definition prevents small indoor fluctuations, within the noise-adjusted background, from being misattributed to cooking emissions under Fully-Opened ventilation conditions, where indoor air directly exchanges with outdoors.

The rise-rate (μg m^–3^ min^–1^) quantifies how rapidly PM_2.5_ concentrations increase during cooking^19^

2 $$\mathrm{rise}‐\mathrm{rate}=\frac{{\Delta \mathrm{PM}}_{\mathrm{peak}}}{t_{\mathrm{peak}}-t_{0}}$$

Where ΔPM_peak_ is defined above, *t*_0_ is the logged cooking start time and *t*_peak_ is the timestamp at which the maximum indoor concentration occurred. during logged cooking period.

The time-normalized PM_2.5_ dose (*D*_PM,norm_) (μg m^–3^) provides a standardized metric for cooking-related exposure by calculating the average, baseline-corrected concentration accumulated per unit time during cooking activities. This normalization enables direct comparison of exposure intensity across cooking episodes of varying durations, eliminating bias from differences in cooking times. It was calculated as^43^

3 $$D_{\mathrm{PM},\mathrm{norm}}=\frac{1}{t_{\mathrm{peak}}-t_{0}}\int_{t_{0}}^{t_{\mathrm{peak}}}\left[C_{\left(t\right)}-C_{\left(0\right)}\right]dt$$

Where *C*_(*t*)_ is the instantaneous PM_2.5_ concentration; *C*_(0)_ is the concentration at the beginning of cooking.

CO_2_ build-up (*D*_CO2,norm_) (ppm) was quantified over the entire cooking period to proxy ventilation efficiency. For each event, with start at *t*_0_ and end at *t*_end_, we set the baseline CO_2(0)_ equal to the mixing ratio at *t*_0_ and then computed^44^

4 $$D_{{\mathrm{CO}}_{2},\mathrm{norm}}=\frac{1}{t_{\mathrm{end}}-t_{0}}\int_{t_{0}}^{t_{\mathrm{end}}}\max\left[{\mathrm{CO}}_{2\left(t\right)}-{\mathrm{CO}}_{2\left(0\right)},0\right]dt$$

Where CO_2(*t*)_ is the indoor CO_2_ level at the end of cooking.

## 3 Results and Discussion

### 3.1 Temporal Patterns of Indoor PM_2.5_ and CO_2_ during the Study Period

Continuous seven-week measurements of PM_2.5_ and CO_2_ were conducted in both the living room/kitchen and bedroom to characterize the indoor air quality dynamics throughout the study period. The resulting temporal patterns are presented in Figure 2, where panel (a) depicts the calibrated indoor PM_2.5_ levels for both rooms (shown as hourly means derived from 5 min measurements), with the red dashed line indicating the WHO air quality guideline level of 15 μg m^–3^ (24 h mean, shown for reference 45) panel (b) shows the corresponding outdoor PM_2.5_ concentrations; and panel (c) presents the indoor CO_2_ mixing ratios in both rooms, with the purple dashed line marking the 1000 ppm threshold commonly associated with insufficient ventilation indoors.^46,47^ The indoor environment’s response to continuous ventilation was illustrated during a representative nonoccupancy period (19 August, 11:00–21 August, 20:00). With all windows open, CO_2_ levels remained consistently low and stable (<450 ppm) throughout this interval, confirming the absence of significant indoor occupant sources. Within noncooking periods, indoor PM_2.5_ concentrations exhibited similar temporal variability to outdoor levels, suggesting a dominant influence of outdoor infiltration when indoor emissions were absent (see Figure S8). The indoor temperatures in R1 and R2 were recorded in Figure S9.

**Figure 2:**
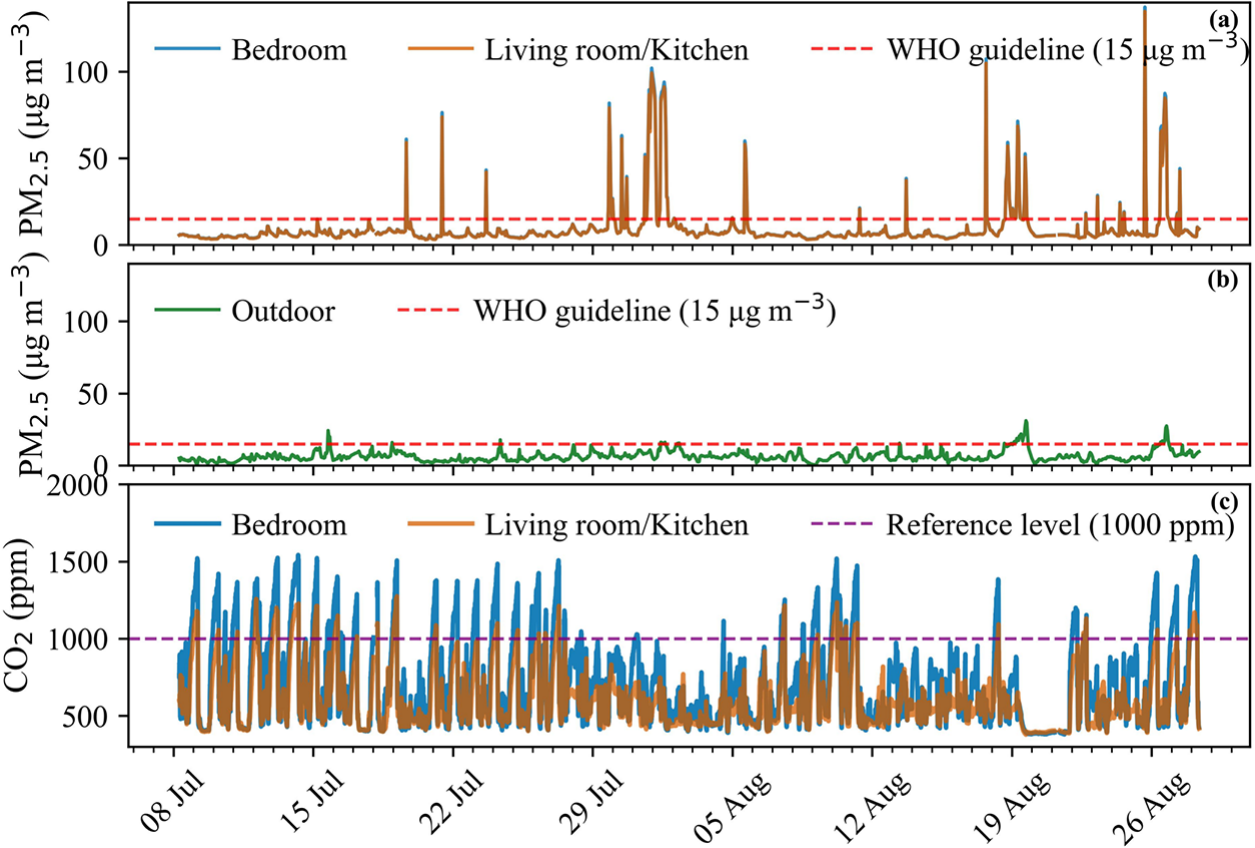
(a, b) Time series of calibrated indoor (bedroom and living room/kitchen) and outdoor PM_2.5_ concentrations during the 7-week monitoring campaign; (c) time series of indoor CO_2_ mixing ratios in the bedroom and living room/kitchen.

Throughout the campaign, indoor PM_2.5_ levels generally tracked outdoor concentrations, particularly during periods of sufficient ventilation as indicated by low indoor CO_2_ levels. Distinct episodic peaks in indoor PM_2.5_ consistently aligned with documented cooking activities (see Table S2). Notably, some of these peaks occurred despite adequate ventilation conditions, indicating that the high emission potential of cooking can overwhelm natural ventilation over short periods, thereby elevating short-term indoor exposure. CO_2_ profiles revealed characteristic accumulation patterns associated with occupancy, exhibiting clear spatial and temporal differences between the bedroom and the living room/kitchen. As evident in Figure 2(c), the bedroom consistently accumulated higher CO_2_ concentrations during nighttime hours, attributable to prolonged occupant presence and respiratory emissions during sleep periods. Subsequently, the living room/kitchen area often showed rising CO_2_ levels following the accumulation in the bedroom, indicating interzonal transport of CO_2_-enriched air between spaces.

Indoor PM_2.5_ concentrations were generally below or near the WHO guideline of 15 μg m^–3^,^45^ with statistical characteristics summarized in Table 1. The distributions were markedly right-skewed, as evidenced by median concentrations substantially lower than the means (6.1 μg m^–3^ vs 9.5 μg m^–3^ in the bedroom; 6.0 μg m^–3^ vs 9.2 μg m^–3^ in the living room/kitchen). The 90th percentile values of 12.0 μg m^–3^ and 11.9 μg m^–3^, respectively, indicate that the highest 10% of measurements approached the WHO guideline, while maximum concentrations reached 137.3 μg m^–3^ and 134.8 μg m^–3^ in the bedroom and the living room/kitchen during extreme pollution events predominantly associated with intensive cooking activities. Such extreme short-term increases are consistent with previous findings that demonstrated the strong emission potential of cooking in enclosed domestic settings, especially under limited ventilation conditions.^18,19,48^ This divergence from outdoor concentrations supports indoor emissions as the primary source of these peaks. Outdoor PM_2.5_ levels were generally lower (mean: 6.3 μg m^–3^, median: 5.6 μg m^–3^), though indoor concentrations exceeded outdoor levels by approximately 45% on average, suggesting significant contributions from indoor sources.

**Table 1:** Statistical Summary of PM_2.5_ Concentrations (μg m^–3^) in the Living Room/Kitchen, Bedroom, and Outdoors during the Monitoring Period

statistical parameter	living room/kitchen	bedroom	outdoor
Mean	9.2	9.5	6.3
Median	6.0	6.1	5.6
10th Percentile	4.1	4.3	2.9
25th Percentile	4.9	5.1	4.1
75th Percentile	7.6	7.8	7.5
90th Percentile	11.9	12.0	10.7
Maximum	134.8	137.3	31.0
Exceedance of WHO Guideline (%)	7.4	7.5	3.6

Analysis of indoor-to-outdoor (I/O) concentration ratios of PM_2.5_, a well-established metric for identifying pollutant sources in indoor environments,^49–51^ suggests a dominant contribution from indoor PM_2.5_ sources (Figure S10). All cooking events analyzed in this study occurred during daytime hours (08:00–20:00). The distributions were characterized by consistently elevated ratios, with mean I/O values of 1.63 and 1.58 for the bedroom and the living room/kitchen, respectively, indicating that indoor concentrations exceeded outdoor levels by approximately 60% on average. Daytime (8:00–20:00) I/O ratios (mean: 1.75 in bedroom, 1.70 in living room/kitchen) substantially exceeded nighttime (20:00–8:00) values (mean: 1.48 in bedroom, 1.44 in living room/kitchen), reflecting the pronounced impact of daytime activities such as cooking. The median I/O ratios further substantiate this pattern, with daytime medians of 1.31 (bedroom) and 1.29 (living room/kitchen) compared to nighttime medians of 1.09 and 1.06, respectively. The right-skewed distributions, evidenced by means exceeding medians and maximum values reaching 32.5 and 31.9 during extreme events, highlight the occurrence of intense, short-duration pollution episodes.

To better disentangle the influence of outdoor conditions, Figure S8 overlays indoor and outdoor PM_2.5_ concentrations within a unified panel. While indoor and outdoor levels tracked closely during periods of low indoor activity, marked divergence occurred during episodic indoor peaks, reinforcing the interpretation that these spikes are primarily driven by indoor sources rather than ambient infiltration. These patterns collectively indicate that indoor sources, particularly during active occupancy periods, likely drive the observed PM_2.5_ peaks, with outdoor infiltration playing a secondary role.

CO_2_ exhibited pronounced spatial and temporal heterogeneity, with detailed statistics provided in Table S3. Mixing ratios were substantially higher and more variable in the bedroom (mean: 709 ppm; median: 606 ppm) compared to the living room/kitchen (mean: 616 ppm; median: 554 ppm). The 90th percentile values of 1176 ppm (bedroom) and 945 ppm (living room/kitchen) underscore the occurrence of substantial accumulation episodes, with the bedroom experiencing more frequent and severe exceedances of the 1000 ppm reference level (16.5% vs 6.7% in the living room/kitchen). These patterns reflect the combined influence of prolonged occupancy and differential ventilation practices between spaces, with the bedroom subject to extended overnight occupancy with limited ventilation. In addition to night-time accumulation, CO_2_ peaks in the living room/kitchen were observed to occur those in the bedroom, consistent with interzonal airflow transporting CO_2_-enriched air from the bedroom during overnight occupancy. These patterns are consistent with expected occupant presence and room connectivity.^52^

### 3.2 Exposure Characteristics Under Different Ventilation Scenarios

During cooking episodes, our analysis primarily focused on PM_2.5_ exposure during the active cooking phase, as this period represents the most intense and direct human exposure near the emission source when occupants are actively engaged with the hob. While we acknowledge that subsequent decay periods also contribute to overall exposure, the peak concentrations during active cooking present the highest acute exposure risk due to proximity to the source. This approach allows us to characterize the maximum exposure scenarios that occupants experience during cooking activities, which is particularly relevant for assessing acute health risks associated with high-concentration, short-duration exposures. The start and end times of each cooking event were manually logged to accurately capture PM_2.5_ variations (Table S2). Pollutant behavior was examined under three representative ventilation configurations typical of residential settings. Under the FO scenario, both windows and internal doors were opened to facilitate maximum air exchange with the outdoors and between rooms. The DO condition allowed inter-room airflow via open internal doors but excluded outdoor exchange. In contrast, the FC scenario sealed both windows and internal doors, minimizing both inter-room and indoor–outdoor ventilation. Key peak characteristics, including PM_2.5_ concentration increments (ΔPM_peak_), rise rates, cooking durations, and the number of monitored cooking events, are summarized in Table 2 for both R1 and R2 under each ventilation scenario.

**Table 2:** PM_2.5_ Peak Concentration Increment (ΔPM_peak_), Rise Rate, and Cooking Duration Under Different Ventilation Strategies in the Living Room/Kitchen (R1) and Bedroom (R2)^a^ ^,^ ^b^

ventilation strategy	room	*n* (events)	ΔPM_peak_ (μg m^–3^)	rise rate (μg m^–3^ min^–1^)	cooking duration (min)
	R2	26	12.8 ± 27.3	0.9 ± 2.1	14.6 ± 4.2
FO					
	R1	26	12.4 ± 26.3	0.9 ± 2.0	14.6 ± 4.2
	R2	20	23.9 ± 67.6	1.9 ± 4.8	15.3 ± 4.1
DO					
	R1	20	23.5 ± 66.8	1.8 ± 4.7	15.3 ± 4.1
	R2	19	19.8 ± 42.1	2.0 ± 4.7	12.1 ± 6.1
FC					
	R1	19	19.2 ± 41.2	1.9 ± 4.6	12.1 ± 6.1

a Values are expressed as mean ± standard deviation. Ventilation strategies include: (1) Fully-Opened (FO): all windows and internal doors open. (2) Doors-Opened & windows-closed (DO): internal doors open; windows closed; and (3) Fully-Closed (FC): all windows and internal doors closed.

b In some cases, the mean ± SD range extends into nonphysically meaningful negative values due to the high variability and skewed nature of the underlying data. These lower bounds are not to be interpreted literally but reflect the dispersion around the mean.

The substantial variability in PM_2.5_ peak increments (ΔPM_peak_) and rise rates, evidenced by standard deviations exceeding mean values, is statistically expected for the right-skewed distributions characteristic of episodic pollution events. This pattern reflects the true heterogeneity of cooking emissions under real-world conditions rather than indicating data quality issues. Previous field and residential studies have consistently reported strong event-to-event variability and right-skewed distributions in cooking-related PM_2.5_ emissions, attributable to variations in source strength and cooking dynamics (e.g.,^19,53^ ), with short-lived but intense peaks often reaching tens to hundreds of μg m^–3^ during common cooking activities.^18^ Importantly, the consistency in relative differences between ventilation scenarios across multiple events (*n* = 19–26 per scenario) supports the robustness of our findings despite this inherent variability, which accurately captures the heterogeneity of real-world cooking conditions and ventilation effectiveness.

Across all scenarios, cooking-generated PM_2.5_ exhibited rapid and substantial dispersion throughout the apartment, consistent with previous findings on the rapid indoor spread of cooking-related PM_2.5_.^19,54,55^ The ΔPM_peak_ and rise rates were consistently comparable between R1 and R2 even under the FC scenario, mirroring the trends observed in FO and DO scenarios. The ΔPM_peak_ in R1 and R2 consistently differed by <0.6 μg m^–3^, and the rise rates by ≤ 0.05 μg m^–3^ min^–1^ under all ventilation settings. This suggests that, despite the apparent physical isolation of rooms when all windows and doors are shut, pollutant levels remained similar across spaces, indicating that physical separation alone may be limited in containing localized emissions.^19,56,57^ The slightly higher PM_2.5_ levels occasionally observed in the bedroom during cooking likely reflect slower local air exchange and longer particle residence times compared to the source room, rather than differences in emission strength or sensor performance.

Among the three scenarios, FO clearly demonstrated the lowest average ΔPM_peak_ and rise rates in both rooms, confirming its superior ability to dilute emissions through enhanced air exchange. Consistent with these observations, previous experimental studies have shown that natural ventilation, including window opening, can substantially reduce cooking-generated PM_2.5_ concentrations compared to restricted-ventilation conditions (e.g.,^48^). Interestingly, the FC scenario did not consistently outperform the DO configuration, despite its seemingly tighter isolation. PM_2.5_ levels under FC were comparable to or even slightly higher than those under DO, likely due to passive inter-room leakage or structural infiltration pathways.^56^ These results indicate that, under the tested conditions, physical sealing alone was not sufficient to prevent pollutant transfer, especially in compact or poorly ventilated layouts.

Moreover, DO exhibited the greatest variability in ΔPM_peak_ and rise rate values, as indicated by the highest standard deviations. This reflects the unstable nature of partial ventilation, which may alternate between pollutant retention and partial dispersion depending on transient factors like pressure gradients or occupant movement. It is also worth noting that PM_2.5_ concentrations varied considerably even under the same ventilation strategy, highlighting the intrinsic variability of real-life cooking emissions.

To further account for ventilation efficiency and exposure duration, we derived time-normalized metrics for both PM_2.5_ and CO_2_, the latter serving as a ventilation proxy. Numerical summaries by room and scenario (*n*, median, Q1 [25th percentile], Q3 [75th percentile], IQR [Q3 – Q1] and mean) are reported in Tables S4–S5. Figure 3 summarizes these metrics across rooms and strategies: panel (a) shows the normalized PM_2.5_ exposure (*D*_PM,norm_), and panel (b) the corresponding normalized CO_2_ accumulation (*D*_CO2,norm_). The corresponding full distributions of event-level values are provided in Figure S11 for completeness.

**Figure 3:**
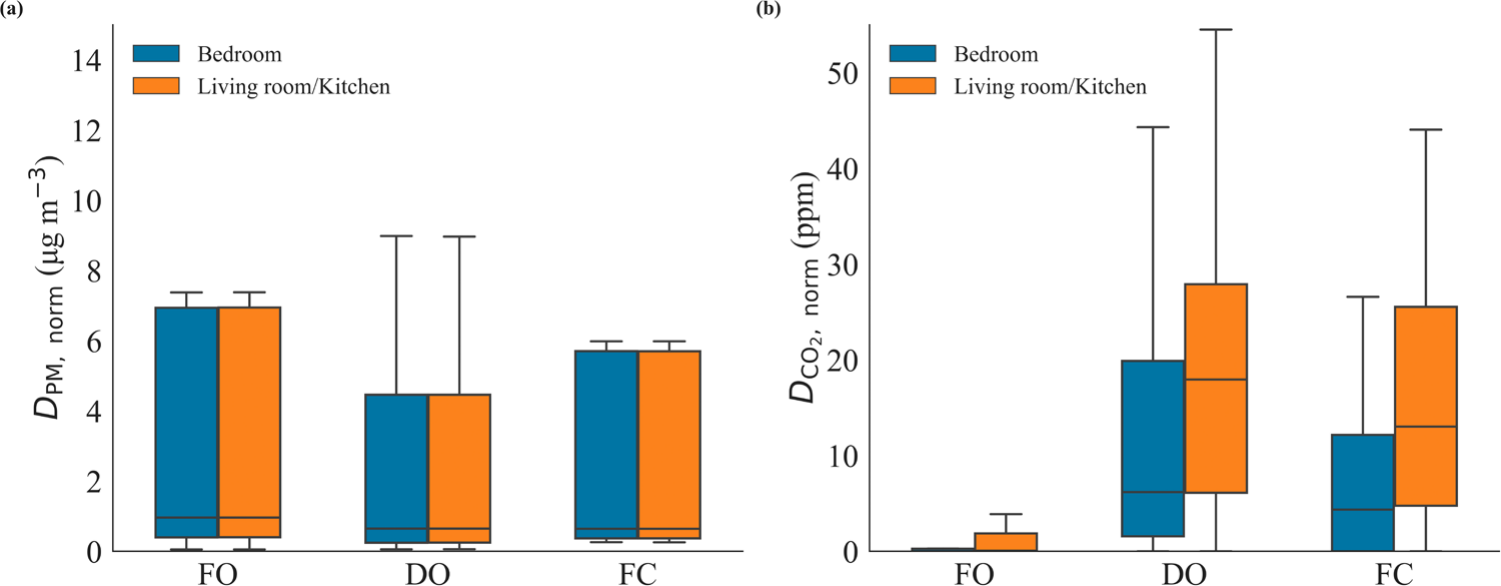
Box plots of time-normalized exposure metrics across three ventilation strategies during cooking events. (a) Time-normalized PM_2.5_ dose (*D*_PM,norm_; μg m^–3^) and (b) Time-normalized CO_2_ accumulation rate (*D*_CO2,norm_; ppm), used as a ventilation proxy. Ventilation strategies include: (1) Fully-Opened (FO): all windows and internal doors open. (2) Doors-Opened & windows-closed (DO): internal doors open; windows closed; and (3) Fully-Closed (FC): all windows and internal doors closed.

Consistent with earlier findings, FO achieved the lowest mean *D*_PM,norm_ across both rooms (14.9 and 15.4 μg m^–3^ in R1 and R2, respectively), reflecting efficient dilution. This represents a significant reduction in PM_2.5_ exposure under FO conditions: 55.8% lower than DO in R2 and 58.4% lower in R1, and 27.9% lower than under FC in R2 and 28.9% lower in R1. Under FO conditions, the mean *D*_PM,norm_ is comparable in magnitude to our operational threshold based on the WHO 24-h guideline (15 μg m^–3^).^45^ This indicates that targeted window opening during cooking can constrain the excess PM_2.5_ to relative health levels and therefore constitutes an effective, low-energy control; by reducing reliance on mechanical ventilation while maintaining indoor air quality, it also aligns with decarbonisation/net-zero objectives.

In contrast, both DO and FC produced markedly higher and more dispersed *D*_PM,norm_ values, indicative of reduced and inconsistent ventilation performance. The DO configuration exhibited a higher frequency of large *D*_PM,norm_ outliers, with heavier upper tails, reflecting substantial pollutant accumulation in the R1 due to the absence of outdoor air exchange. Elevated values were also observed in the R2, pointing to rapid inter-room transport of cooking-generated PM_2.5_ through the open internal door. This suggests the role of R1 as the primary emission source and highlights how internal connectivity, despite external isolation, can facilitate pollutant propagation across spaces. In contrast, under the FC condition, both doors and windows were shut, physically isolating rooms. Yet, the presence of elevated *D*_PM,norm_ in the R2 suggests that full enclosure offers limited effectiveness in containing PM_2.5_. These findings imply that passive leakage and structural permeability could contribute to pollutant transfer, although this was not directly quantified in the present study.

To corroborate these patterns, in panel (b), CO_2_ accumulation (*D*_CO2,norm_) exhibits the expected ventilation gradient and consistent patterns with PM_2.5_. Under the FO condition, CO_2_ mixing ratios remained low in both rooms, with very narrow quartile ranges (R2: median 0.0 ppm [Q1–Q3:0.0–0.3]; R1:0.07 ppm [0.0–1.9]) (see Table S5). These low Q1 and tight IQRs indicate effective real-time exchange with outdoor air and minimal indoor accumulation. In contrast, during DO events, the quartiles shift sharply upward—R1 shows Q1–Q3 = 6.1–27.9 ppm versus 1.6–19.9 ppm in R2—consistent with CO_2_ being generated in the source zone (R1) during cooking and progressively accumulating because of restricted ventilation. The internal door facilitated inter-room transfer, leading to elevated values also in R2. The R1 showed a much higher density of data points around 20 ppm, clearly exceeding the corresponding R2 values and confirming its role as the primary source zone. Under the FC strategy, CO_2_ build-up was even more pronounced in R1, with faster and larger accumulations than in R2. This suggests that in the absence of any active ventilation paths, CO_2_, and by extension, other indoor pollutants, tend to concentrate at the emission source and gradually infiltrate into adjacent spaces via passive leakage, in limited quantities.

These results reinforce the alignment between short-term PM_2.5_ exposure and CO_2_-based ventilation proxies. The convergence of these independent metrics supports the integrity of the experimental design and supports the use of CO_2_ as a real-time surrogate for ventilation efficiency. Furthermore, the stark contrast between FO and the other configurations highlights the importance of outdoor connectivity for reducing indoor pollutant levels under similar conditions.

### 3.3 Research Implications

This study applied a multimetric framework (ΔPM_peak_, rise rate, and *D*_PM,norm_) for capturing short-term cooking exposure dynamics. The alignment between PM_2.5_ reductions and CO_2_-based ventilation indicators supports using CO_2_ as a real-time ventilation proxy in similar field studies, while recognizing that CO_2_ traces ventilation, not particulate sources, per se.

These findings indicate that, in this case-study context (a modern, naturally ventilated UK apartment during summer), ventilation practices during cooking episodes influence short-term PM_2.5_ exposure. The FO scenario produced the lowest PM_2.5_ peaks and the lowest time-normalized exposure across rooms. Quantitatively, FO reduced mean *D*_PM,norm_ by approximately 56–58% relative to DO and by 28–29% relative to FC (R1/R2) and reduced mean ΔPM_peak_ by approximately 46–47% relative to DO and ∼35% relative to FC (Table 2). These results support intentional, short-duration window opening during cooking as an effective, low-energy measure to reduce acute PM_2.5_ exposure.

Implementing FO in real homes must balance security, thermal comfort, and energy consumption. In urban settings, safety concerns may limit window opening; secure window limiters, trickle/secure vents, or timed opening aligned with the highest-emission steps (e.g., frying, searing) are pragmatic options. Seasonal factors also matter. This study was conducted during summer, when indoor–outdoor temperature differences are typically smaller and buoyancy-driven ventilation is relatively weak. In winter, larger indoor–outdoor temperature differences can increase buoyancy-driven flow (stack effect),^30^ potentially enabling shorter, more efficient bursts of ventilation to achieve comparable air exchange. Context-specific recommendations should therefore account for climate, dwelling layout, and occupant constraints, and merit targeted evaluation.

From a public health perspective, the consistent exposure reductions observed under FO support practical messaging aligned with existing guidance: use local extraction where available and increase short-term ventilation during cooking (e.g., opening windows and, where feasible, enabling cross-ventilation), while considering outdoor air quality and safety constraints.^58^ For housing providers and designers, the results highlight the value of secure, energy-efficient ventilation options near cooking zones that facilitate outdoor air exchange during high-emission activities.

Findings derive from a single apartment, summer period, and electric hob with no mechanical exhaust in operation; generalization to other building types, seasons, and fuel/appliance mixes (e.g., gas, range hoods in use) requires caution. While inter-room transport during active cooking was explicitly examined in this study, postcooking decay phases were not included in the exposure metrics and may contribute to additional time-integrated exposure across rooms. In addition, outdoor PM_2.5_ was characterized using data from a nearby roadside monitoring station; while this may overestimate absolute background concentrations, it is unlikely to affect the relative indoor–outdoor contrasts and scenario comparisons that form the focus of this study. Future studies should (i) test seasonal performance and optimize burst-ventilation durations; (ii) evaluate security-constrained designs that maintain airflow; and (iii) examine behavioral adoption and persistence in diverse households, (iv) incorporate on-site or near-residence outdoor PM2.5 measurements to obtain more accurate characterization of local outdoor concentrations. These steps will help convert the observed benefits of simple, occupant-controlled ventilation into practical, year-round guidance.

## 4 Conclusions

This study shows that occupant-controlled natural ventilation during cooking materially reduces short-term PM_2.5_ exposure in a modern, naturally ventilated UK apartment. Across 19–26 cooking events per scenario, the fully opened (FO: all windows and internal doors open) scenario consistently produced the lowest time-normalized exposure (achieving mean concentrations of 14.9 μg m^–3^ in the living room/kitchen and 15.4 μg m^–3^ in the bedroom) and the smallest peaks, reducing PM_2.5_ concentrations by approximately 57% versus DO (internal doors open; windows closed) and 28% relative to FC (all windows and internal doors closed). Exposure patterns were similar across rooms even when doors and windows were closed, indicating rapid inter-room transport and that outdoor air exchange (not physical separation) governs short-term outcomes. CO_2_-based ventilation proxies (*D*_CO2,norm_) tracked these gradients, supporting CO_2_ as a practical real-time indicator of ventilation effectiveness in this setting. While results are limited to a single dwelling, summer conditions, and an electric hob without mechanical exhaust, they demonstrate that short, intentional windows opening during high-emission cooking steps is an immediately actionable, low-energy strategy to curb acute PM_2.5_ exposure.

## Supplementary Material



## Data Availability

The data sets used and/or analyzed during the current study are available from the corresponding author on reasonable request.

## Notes

The Supporting Information provides an overview of the study location, and the low-cost air quality sensors used in the field campaign (Figures S1 and S2); the section Sensor Calibration and Performance Evaluation presents the calibration procedures for the low-cost sensor used in this study, including colocation experiments, piecewise multivariate regression calibration, and postcalibration performance assessment (Figures S3–S7); the SI also presents additional contextual results, including time series of indoor and outdoor PM_2.5_, indoor temperature variation, and distributions of indoor-to-outdoor PM_2.5_ ratios (Figures S8–S10), summarizes the distributions of time-normalized PM_2.5_ dose and CO_2_ accumulation metrics under different ventilation strategies during cooking events (Figure S11), summarizes the geometric and ventilation-related characteristics of the experimental apartment (Table S1), lists the cooking events and corresponding ventilation scenarios analyzed in this study (Table S2), provides statistical summaries of indoor CO_2_ mixing ratios (Table S3) and presents time-normalized PM_2.5_ and CO_2_ metrics, respectively, stratified by ventilation scenario and room (Tables S4 and S5) (PDF)
