# Supplementary material for: Natural Ventilation Reduces Cooking-Related PM2.5 Peaks Indoors
Source: ACS EST Air. 2026 Jan 29;3(2):590–9. doi: 10.1021/acsestair.5c00427 (PMC12910602; doi:10.1021/acsestair.5c00427)
Supplement: Supplementary file 1 [file ea5c00427_si_001.pdf]

Supporting Information

Natural Ventilation Reduces Cooking-related $PM_{2.5}$ Peaks indoors

Yizhou Su[1], Yuqing Dai[1,*], Zongbo Shi[1], Yirui Jiang[2], Lingchen Kong[3], and Christian Pfrang[1,4,*]

[1]School of Geography, Earth and Environmental Sciences, University of Birmingham, Edgbaston, B15 2TT, Birmingham, UK

[2]Department of Computer Science and Technology, University of Cambridge, Cambridgeshire, CB3 0FD, UK

[3]Department of Architecture, University of Cambridge, Cambridge, CB2 1PX, UK

[4]Department of Meteorology, University of Reading, Whiteknights, Earley Gate, RG6 6BB, Reading, UK

*Corresponding authors: y.dai.2@bham.ac.uk and c.pfrang@bham.ac.uk

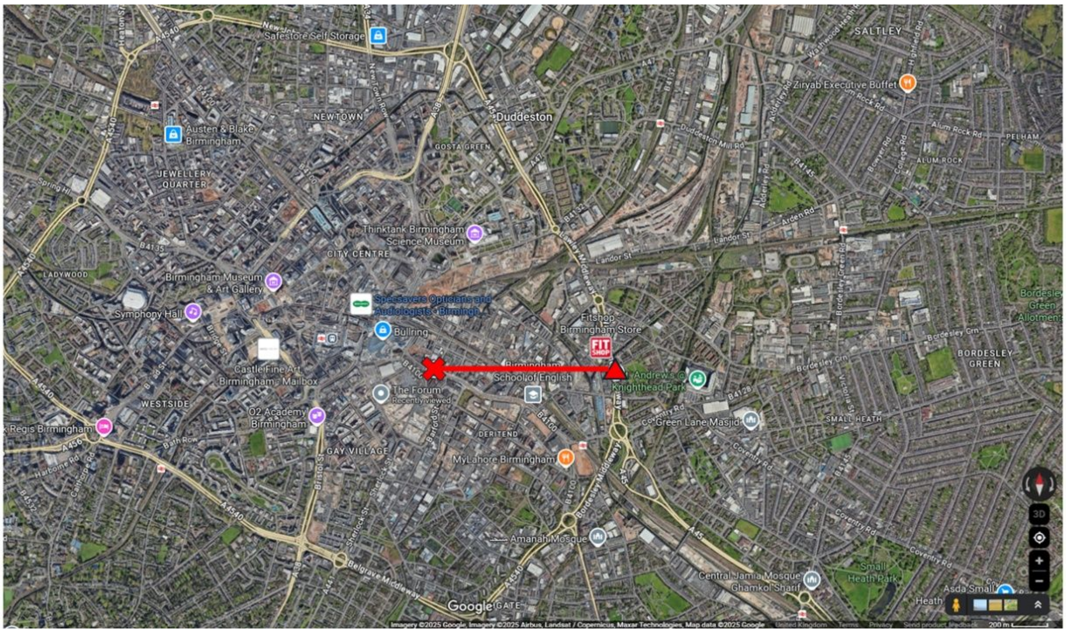

Figure S1. Map of Birmingham city centre showing the studied apartment (red ×) and the outdoor $PM_{2.5}$ monitoring station (red ▲, AURN A4540, Keeley Street). The straight-line distance between the two locations is approximately 1.34 km (0.83 miles).

(Map data ©2025 Google, https://www.google.com/maps)

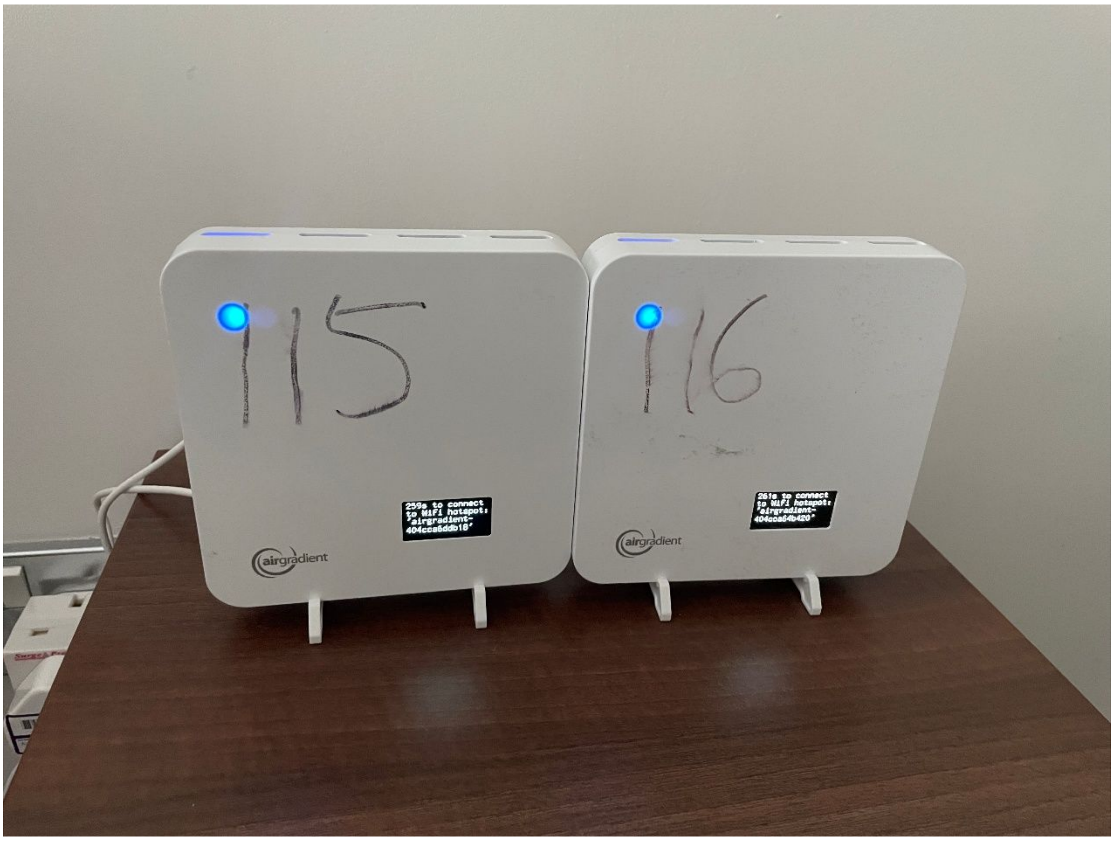

Figure S2. Photo of the two AirGradient ONE $PM_{2.5}$ monitors used in the study. Both were laboratory-calibrated against a Palas Fidas® 200E prior to deployment. During field measurements, they were placed centrally in the living room and bedroom, respectively, to capture representative indoor concentrations.

(https://www.airgradient.com/indoor)

(Photo by the author)

Sensor Calibration and Performance Evaluation

Given that optical light-scattering based low-cost $PM_{2.5}$ sensors are sensitive to environmental conditions such as RH and T, primarily due to hygroscopic particle growth, which alters scattering intensity [1], we calibrated air quality sensors' RH and T outputs against the reference instrument [2]. The calibration results are shown in Figure S3, where both bedroom and kitchen sensors exhibited strong linear agreement with reference RH and T values, with R^2 values exceeding 0.94 for RH and 0.99 for T. These calibrated RH and T values were subsequently incorporated as predictors in the $PM_{2.5}$ multivariate regression model.

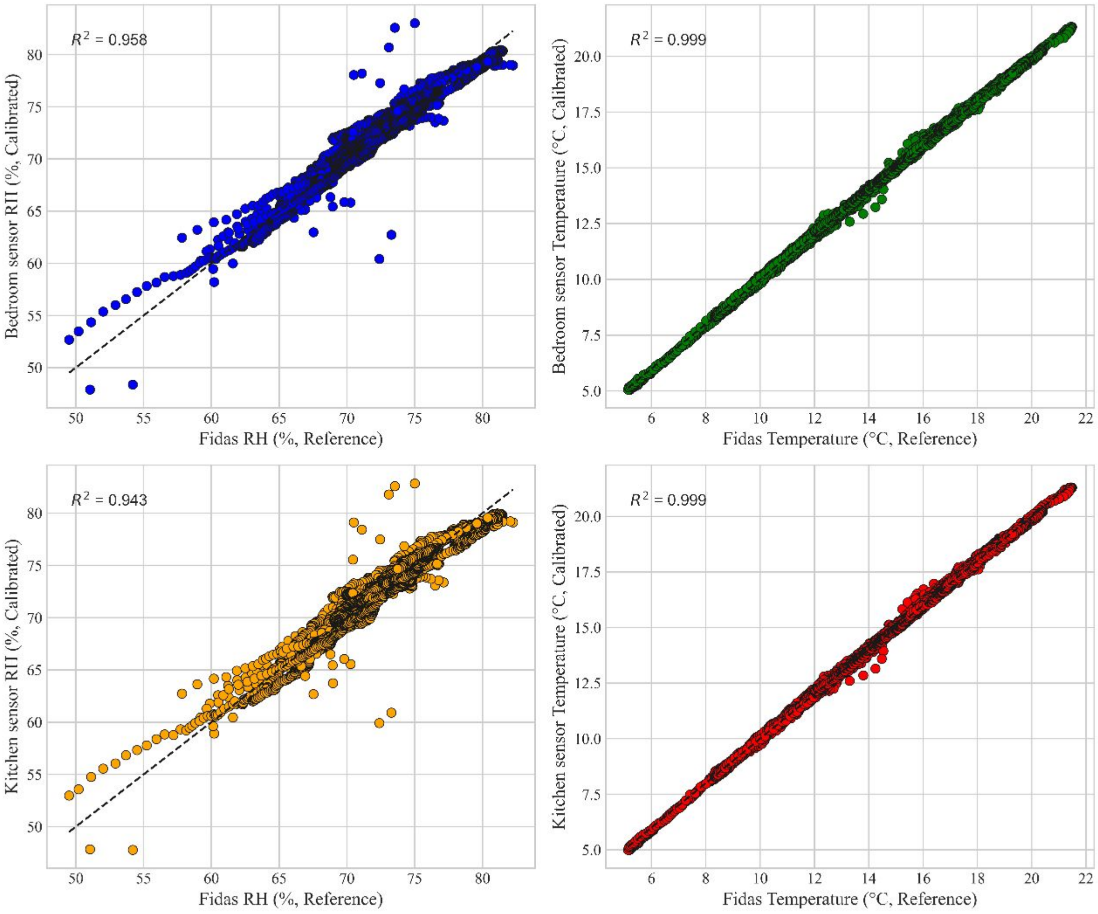

Figure S3. Calibration results for relative humidity (RH) and temperature (T) of the two AirGradient sensors against the Palas Fidas® 200 E reference instrument.

Figure S4 shows the results of co-location measurements with the Fidas unit, which were used to evaluate the sensor's performance in measuring $PM_{2.5}$ mass concentration. Figure S4(a) shows strong agreement between the two sensors themselves, indicating their high internal consistency. However, as depicted in Figures S4(b-c), comparison with the Fidas revealed distinct deviations from the 1:1 line, with systematic underestimation at lower concentrations (≤ 20 μg m^{-3}) and overestimation at higher concentrations. This pattern aligns with previously documented non-linearities in low-cost optical sensor responses, often linked to humidity and aerosol loading effects [3].

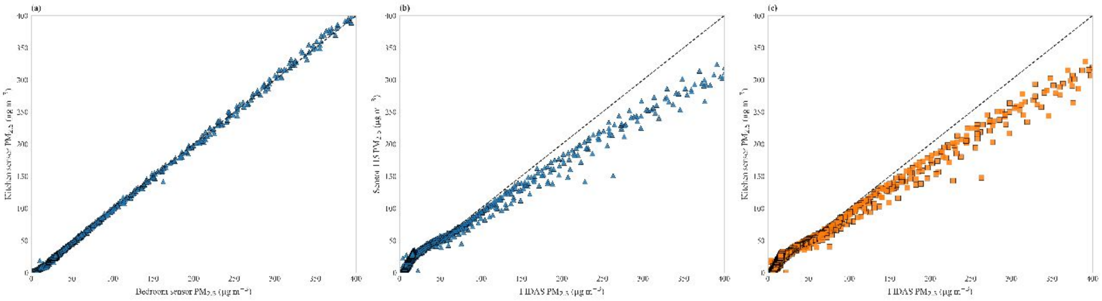

Figure S4. Sensor co-location comparisons prior to calibration. (a) agreement between bedroom and kitchen $PM_{2.5}$ sensors; (b–c) raw sensor values from kitchen and bedroom compared with Fidas reference.

In light of these observations, a two-segment piecewise multivariate linear regression approach was applied, stratified by a $PM_{2.5}$ breakpoint of 20 μg m^{-3}. The calibration functions derived for both bedroom and kitchen sensors under the two concentration ranges are summarised in Figure S5. The resulting regression models significantly improved agreement with reference measurements across the full concentration range, as demonstrated in Figure S6, where R^2 values approached or exceeded 0.95, indicating strong model performance. This calibration framework enabled the reliable capture of both background indoor $PM_{2.5}$ levels and the sharp

concentration spikes associated with episodic sources such as cooking.

Sensor (Bedroom) Calibration Models

Low (0-20): FIDAS = 0.482·PM + -0.055·Temp + -0.078·RH + 8.853

High (20-400): FIDAS = 1.147·PM + 0.576·Temp + -0.142·RH + -3.996

Sensor (Kitchen) Calibration Models

Low (0-20): FIDAS = 0.540·PM + -0.016·Temp + -0.079·RH + 8.881

High (20-400): FIDAS = 1.065·PM + 0.510·Temp + -0.166·RH + 8.720

Figure S5. Piecewise multivariate calibration functions applied to sensor data.

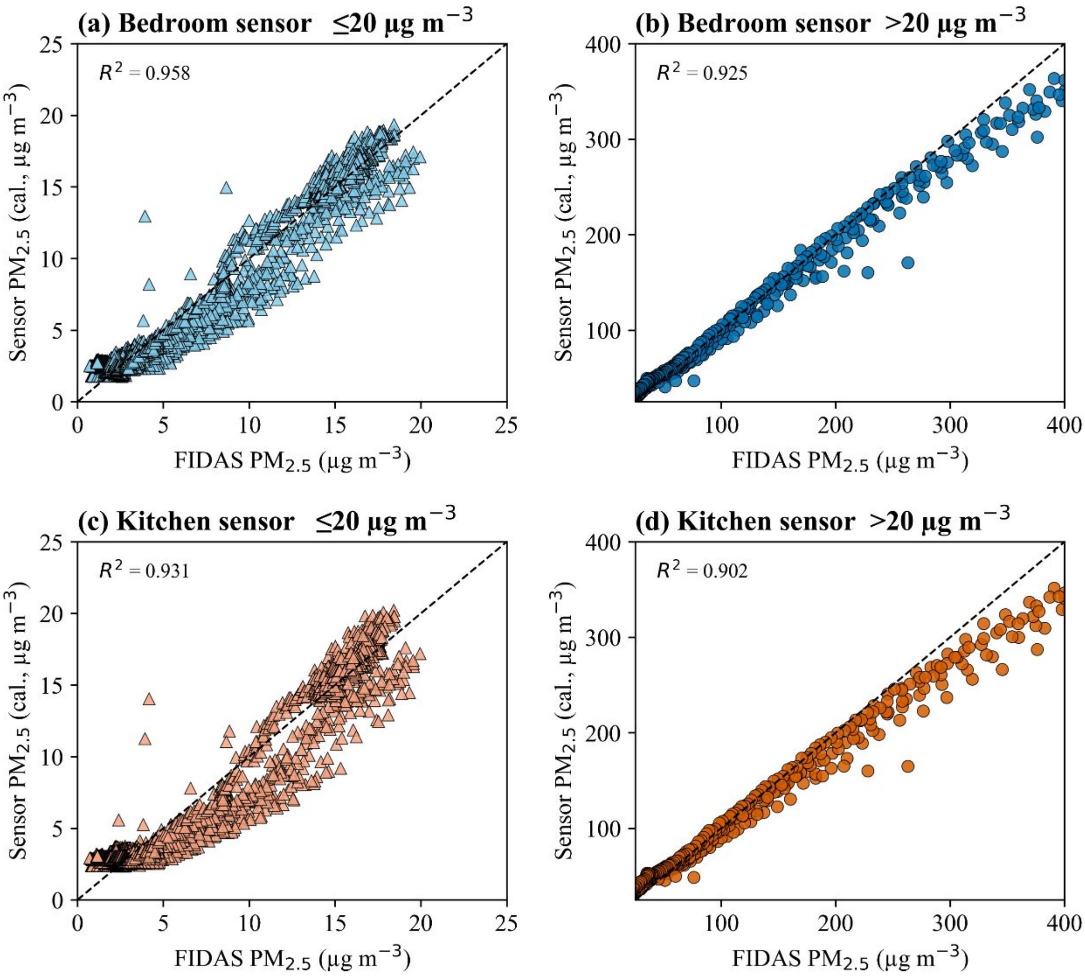

Figure S6. Post-calibration performance of bedroom and kitchen $PM_{2.5}$ sensors against reference Fidas measurements. (a–b) Bedroom sensor performance under low (≤ 20 μg m^{-3}) and high (20–400 μg m^{-3}) concentration ranges; (c–d) Kitchen

sensor performance under corresponding ranges. Calibration conditions spanned RH 48–83% and temperature 5–21 °C.

As shown in Figure S7, the two sensors produced highly concordant CO_2 readings (R^2 = 0.90), with a near 1:1 relationship. This high degree of agreement confirms the stability and internal consistency of the sensors, supporting their use for tracking indoor CO_2 variation and assessing ventilation through temporal mixing ratio changes.

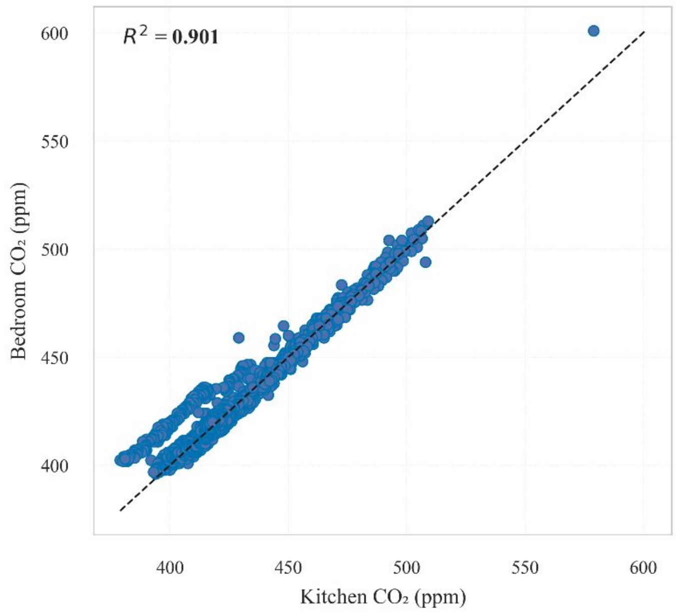

Figure S7. Co-location comparison of CO_2 concentrations between kitchen and bedroom sensors.

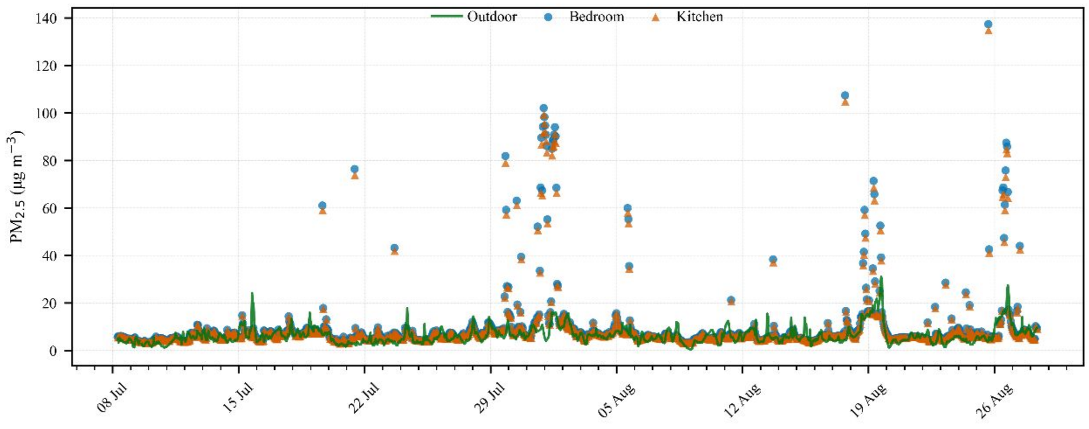

Figure S8. Overlay of calibrated indoor (bedroom and kitchen) and outdoor $PM_{2.5}$ concentrations over the full monitoring period. Periods dominated by cooking-related indoor emissions are characterised by sharp indoor peaks, whereas during non-cooking periods indoor $PM_{2.5}$ varies smoothly and follows outdoor temporal trends.

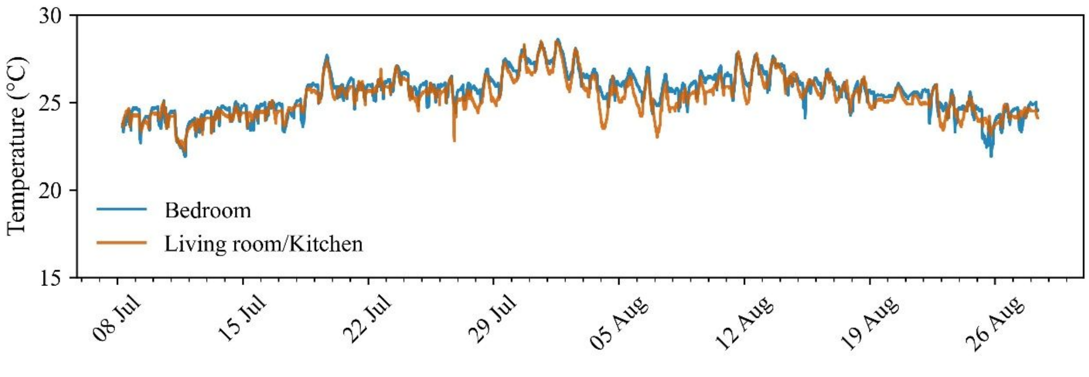

Figure S9. Time series of indoor air temperature measured in the bedroom (R2) and living room/kitchen (R1) over the full monitoring period.

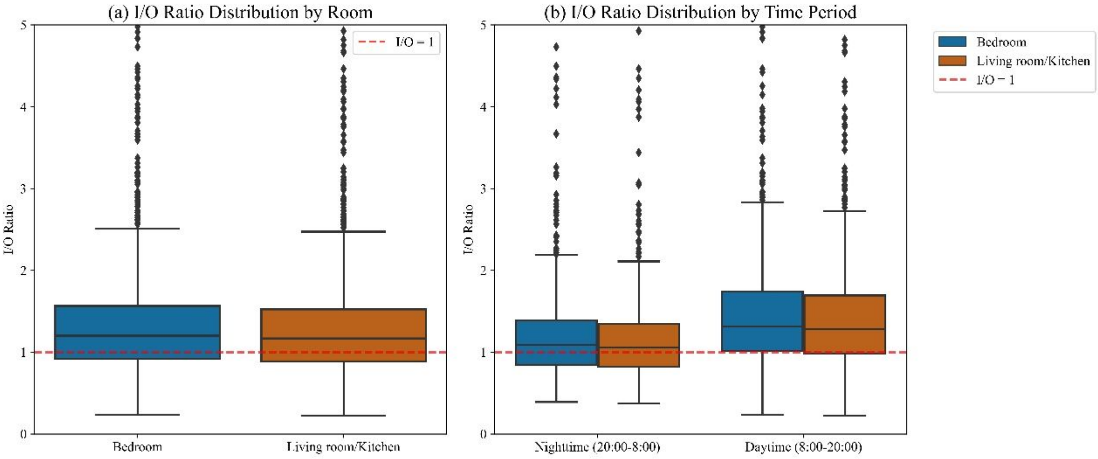

Figure S10. Distribution of indoor-to-outdoor (I/O) $PM_{2.5}$ concentration ratios for (a) the bedroom and living room/kitchen, and (b) by time (daytime: 8:00-20:00, nighttime: 20:00-8:00).

The red dashed line indicates I/O = 1, with values above this threshold indicating dominance of indoor sources. The boxplots show the median (central line), interquartile range (box), 1.5× interquartile range (whiskers), and outliers (points). Higher median I/O ratios and greater variability during daytime hours reflect the substantial impact of indoor activities, particularly cooking, on indoor $PM_{2.5}$ concentrations. Consistently elevated I/O ratios across both rooms and time periods underscore the significant contribution of indoor emission sources to occupant exposure.

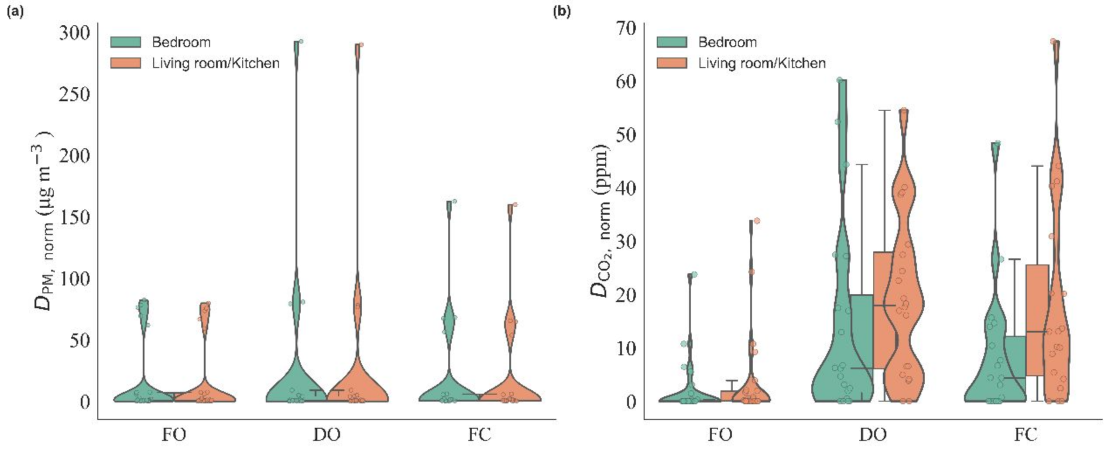

Figure S11. Distribution of time-normalised exposure metrics across three ventilation strategies during cooking events. (a) Time-normalised $PM_{2.5}$ dose ($D_{PM,norm}$; μg m^{-3}) and (b) Time-normalised CO_2 accumulation rate ($D_{CO2,norm}$; ppm), used as a ventilation proxy. Ventilation strategies include: (1) Fully-Opened (FO): all windows and internal doors open. (2) Doors-Opened & windows-closed (DO): internal doors open; windows closed; and (3) Fully-Closed (FC): all windows and internal doors closed. Violins show the distribution of event-level values; boxes indicate medians and interquartile ranges; points are individual events.

Table S1. Geometric and ventilation-related characteristics of the living room/kitchen (R1), bedroom (R2), and corridor (R3) of experimental apartment, including floor area, ceiling height, room volume, and effective opening areas of windows (W1, W2) and internal doors (D1, D2).

	Floor area (m^2)	Volume (m^3)	Effective ventilation area (m^2)		Ceiling Height (m)
			Window	Door	
R1	18.87	44.35	0.33	1.64	2.35
R2	11.28	26.50	0.15	1.64	2.35
R3	6.72	15.79	/	1.64	2.35

Table S2. Summary of experimental ventilation scenario and cooking periods. Ventilation scenarios include: (1) Fully-Opened (FO): all windows and internal doors open. (2) Doors-Opened & windows-closed (DO): internal doors open; windows closed; and (3) Fully-Closed (FC): all windows and internal doors closed.

Start	End	Scenario	$PM_{2.5}$out (μg m^{-3})
08/07/2024 12:30	08/07/2024 12:45	FO	3.9
08/07/2024 16:20	08/07/2024 16:40	FO	3.3
09/07/2024 08:20	09/07/2024 08:45	FO	5.1
09/07/2024 18:30	09/07/2024 18:50	FO	1.7
10/07/2024 08:20	10/07/2024 08:35	FO	2.7

10/07/2024 12:00	10/07/2024 12:20	DO	2.9
10/07/2024 16:40	10/07/2024 17:00	FO	2.4
11/07/2024 07:35	11/07/2024 07:45	FO	3.5
11/07/2024 20:45	11/07/2024 21:05	DO	2.6
12/07/2024 07:40	12/07/2024 08:00	FO	6.7
12/07/2024 13:30	12/07/2024 13:40	FO	5.2
12/07/2024 17:15	12/07/2024 17:30	FO	8.3
13/07/2024 10:25	13/07/2024 10:35	FO	5.7
13/07/2024 18:10	13/07/2024 18:35	DO	4.2
14/07/2024 08:55	14/07/2024 09:15	DO	4.1
14/07/2024 12:05	14/07/2024 12:25	DO	4.6
14/07/2024 19:05	14/07/2024 19:15	FC	4.4
15/07/2024 09:00	15/07/2024 09:15	FO	8.4
15/07/2024 12:30	15/07/2024 12:45	DO	5.8
15/07/2024 18:55	15/07/2024 19:10	FC	24.2
16/07/2024 07:00	16/07/2024 07:15	FC	6.3
16/07/2024 13:00	16/07/2024 13:15	FC	6.7
16/07/2024 17:30	16/07/2024 17:45	DO	6.0
17/07/2024 07:40	17/07/2024 07:45	FC	10.0
17/07/2024 12:45	17/07/2024 13:00	FO	6.1
17/07/2024 18:50	17/07/2024 19:05	DO	6.8
18/07/2024 07:40	18/07/2024 07:50	FO	9.2
18/07/2024 12:10	18/07/2024 12:20	FO	6.5
18/07/2024 18:20	18/07/2024 18:35	FO	5.6

19/07/2024 11:05	19/07/2024 11:20	DO	8.2
19/07/2024 16:05	19/07/2024 16:25	DO	6.6
20/07/2024 10:25	20/07/2024 10:40	FO	2.3
20/07/2024 19:35	20/07/2024 19:50	DO	2.8
29/07/2024 16:55	29/07/2024 17:20	FC	5.2
30/07/2024 10:30	30/07/2024 10:45	FC	4.6
30/07/2024 16:40	30/07/2024 16:55	FO	7.3
31/07/2024 06:30	31/07/2024 06:45	FC	8.5
31/07/2024 18:35	31/07/2024 18:45	FC	8.7
01/08/2024 07:50	01/08/2024 08:10	FO	10.9
01/08/2024 16:30	01/08/2024 16:45	FO	14.2
02/08/2024 19:35	02/08/2024 19:50	FC	5.6
04/08/2024 13:40	04/08/2024 14:00	FO	6.0
05/08/2024 15:10	05/08/2024 15:40	FC	10.4
05/08/2024 17:10	05/08/2024 17:30	FO	6.6
07/08/2024 09:00	07/08/2024 09:15	DO	5.9
07/08/2024 11:40	07/08/2024 12:00	DO	4.9
09/08/2024 17:55	09/08/2024 18:10	FO	3.4
11/08/2024 10:40	11/08/2024 10:50	FC	5.4
12/08/2024 15:50	12/08/2024 16:05	FC	9.2
13/08/2024 17:25	13/08/2024 17:45	FO	5.2
14/08/2024 16:25	14/08/2024 16:35	FC	6.1
14/08/2024 19:10	14/08/2024 19:25	FC	5.3
15/08/2024 20:05	15/08/2024 20:15	DO	8.8

16/08/2024 12:00	16/08/2024 12:15	DO	4.4
16/08/2024 18:20	16/08/2024 18:35	DO	4.0
17/08/2024 06:35	17/08/2024 06:45	DO	4.2
17/08/2024 17:00	17/08/2024 17:25	FC	4.2
18/08/2024 17:15	18/08/2024 17:30	FC	13.5
22/08/2024 17:30	22/08/2024 17:45	FO	2.8
23/08/2024 21:55	23/08/2024 22:15	DO	5.7
24/08/2024 10:30	24/08/2024 10:50	DO	5.0
24/08/2024 17:45	24/08/2024 17:55	FC	2.5
25/08/2024 06:55	25/08/2024 07:05	FC	5.3
25/08/2024 15:55	25/08/2024 16:15	DO	3.6
26/08/2024 07:05	26/08/2024 07:15	FO	13.1

Table S3. Statistical summary of CO_2 mixing ratios (ppm) during the monitoring period in the living room/kitchen (R1), and the bedroom (R2).

Statistical Parameter	R1	R2
Mean	616	709
Median	554	606
10th Percentile	433	424
25th Percentile	472	486
75th Percentile	682	873
90th Percentile	945	1176
Maximum	1275	1542
Exceedance of Reference level (%)	6.7	16.5

Table S4. Time-normalised $PM_{2.5}$ ($D_{PM,norm}$; µg m^{-3}) by ventilation scenario and room. Ventilation scenarios include: (1) Fully-Opened (FO): all windows and internal doors open. (2) Doors-Opened & windows-closed (DO): internal doors open; windows closed; and (3) Fully-Closed (FC): all windows and internal doors closed.

Scenario	Room	n	Median	Q1–Q3	IQR	Mean
FO	Bedroom	26	1.0	0.4–6.9	6.5	15.4
FO	Kitchen	26	1.0	0.4–7.0	6.6	14.9
DO	Bedroom	20	0.7	0.3–4.5	4.2	24.0
DO	Kitchen	20	0.7	0.3–4.5	4.2	23.6
FC	Bedroom	19	0.6	0.4–5.7	5.3	19.7
FC	Kitchen	19	0.6	0.4–5.7	5.3	19.2

Note: Q1 is the 25th percentile, Q3 is the 75th percentile; IQR = interquartile range (25th–75th percentile); n indicates the number of events.

Table S5. Time-normalised CO_2 ($D_{CO2, norm}$; ppm) by ventilation scenario and room. Ventilation scenarios include: (1) Fully-Opened (FO): all windows and internal doors open. (2) Doors-Opened & windows-closed (DO): internal doors open; windows closed; and (3) Fully-Closed (FC): all windows and internal doors closed.

Scenario	Room	n	Median	Q1–Q3	IQR	Mean
FO	Bedroom	26	0.0	0.0–0.3	0.3	2.0
FO	Kitchen	26	0.1	0.0–1.9	1.9	3.4
DO	Bedroom	20	6.2	1.6–19.9	18.3	14.5
DO	Kitchen	20	18.0	6.1–27.9	21.8	19.5
FC	Bedroom	19	4.4	0.0–12.2	12.2	8.2
FC	Kitchen	19	13.0	4.8–25.6	20.8	18.2

Note: Q1 is the 25th percentile, Q3 is the 75th percentile; IQR = interquartile range (25th–75th percentile); n indicates the number of events.

References

1. Jayaratne, R., et al., *The influence of humidity on the performance of a low-cost air particle mass sensor and the effect of atmospheric fog.* Atmos. Meas. Tech., 2018. **11**(8): p. 4883-4890.
2. Qian, J., et al., *Enhancing Accuracy of Indoor Air Quality Sensors via Automated Machine Learning Calibration.* EGUsphere, 2025. **2025**: p. 1-20.
3. Feenstra, B., et al., *Performance evaluation of twelve low-cost PM2.5 sensors at an ambient air monitoring site.* Atmospheric Environment, 2019. **216**: p. 116946.
